# The prison-to-community transition as a neuropeptide stress test: an allostatic continuity agenda for addiction care

**DOI:** 10.3389/adar.2026.17562

**Published:** 2026-10-14

**Authors:** Aina Afini Abd Basir, Zalmizy Hussin

**Affiliations:** School of Applied Psychology, Social Work and Policy, College of Arts and Sciences, Universiti Utara Malaysia, Sintok, Malaysia

**Keywords:** addiction, allostasis, community re-entry, incarceration, neuropeptides

## Introduction

In correctional settings, substance use care is often evaluated on the basis of observed abstinence or reduced use during custody, treatment attendance, readiness for change, and motivation to sustain recovery. Yet constrained access to substances does not demonstrate that stress and reward regulation have recovered. Custody may involve uncertainty, limited autonomy, disrupted sleep, and social disconnection; release reintroduces drug-associated cues while adding pressures related to housing, stigma, income, and unstable treatment access. These phases are commonly managed as separate administrative episodes, although they may constitute a single biologically and socially continuous transition.

We argue that the prison-to-community transition should be investigated as a neuropeptide-sensitive allostatic sequence. We define the “carceral allostatic amplifier” as an exposure-linked, within-person worsening of stress-regulatory phenotypes across custody and re-entry, potentially accompanied by coordinated changes in candidate neuropeptide measures. It is a conditional and falsifiable construct, not a biological label: it predicts neither uniform harm across institutions nor a common trajectory for all incarcerated people. Its purpose is to identify when abrupt environmental change, interrupted care, and preexisting dysregulation may converge.

We are unaware of prospective human studies that repeatedly measure neuropeptide trajectories from custody through community re-entry. This Opinion therefore integrates evidence from stress-related addiction neurobiology [1–6] and post-release vulnerability [7–11] to define a testable research agenda. It should not be interpreted as evidence that incarceration produces a specific peptide profile.

## Why observed non-use may conceal allostatic vulnerability

Addiction involves adaptations in reward circuitry and recruitment of stress processes in the extended amygdala. CRF, dynorphin–kappa opioid receptor, and orexin signaling form the primary stress-promoting candidates in this framework; NPY, nociceptin, and context-dependent oxytocin are counter-regulatory candidates [1–6]. Their roles vary by receptor, circuit, substance, and addiction stage. Peripheral concentrations are indirect and cannot be equated with synaptic release, receptor activity, or signaling within a specific brain circuit.

The framework is transdiagnostic at the level of stress regulation, but it should not be assumed to operate identically across substances or populations. Alcohol, opioid, stimulant, and polysubstance use disorders require stratified analyses, as do co-occurring trauma, mental illness, chronic pain, sex-related biology, age, and treatment exposure. Opioid use disorder offers a pragmatic initial test because correctional-care evidence is strongest for medication continuity and post-release outcomes [8–10, 12–14]; findings should not be generalized automatically to other disorders.

Risk is concentrated immediately after release; recent linked data show particularly high opioid-overdose rates during the first 2 weeks [11]. A program assessed only at discharge may therefore appear successful before community cues and structural adversity have tested what was achieved in custody [7–9, 11]. Return to use may then be attributed to motivation alone, even though vulnerability may have accumulated before release. Follow-up should align with the transition rather than administrative convenience.

## A contextual network, not a single biomarker

The prospect of a single blood marker for relapse is attractive but premature. Plasma, serum, saliva, and other peripheral specimens are not interchangeable, and their concentrations may not represent central signaling. Circadian timing, nutrition, medication, acute stress, sex-related biology, specimen handling, extraction, storage, and assay procedures can influence results. Oxytocin illustrates this uncertainty: peripheral measurement is methodologically sensitive, and a recent trial found no added benefit of intranasal oxytocin beyond alcohol behavioral couple therapy [15–18].

We therefore propose a within-person network hypothesis. Under sustained adversity, greater recruitment of stress-promoting systems together with insufficient counter-regulation may accompany hyperarousal, poor sleep, reduced social safety, stress-reactive craving, and diminished recovery motivation. Housing, stigma, relationships, income, and treatment access are not background “noise” or nuisance covariates; depending on the causal question, they may be exposures, moderators, mediators, or outcomes. Figure 1 depicts hypothesized associations rather than an established causal pathway.

**FIGURE 1:**
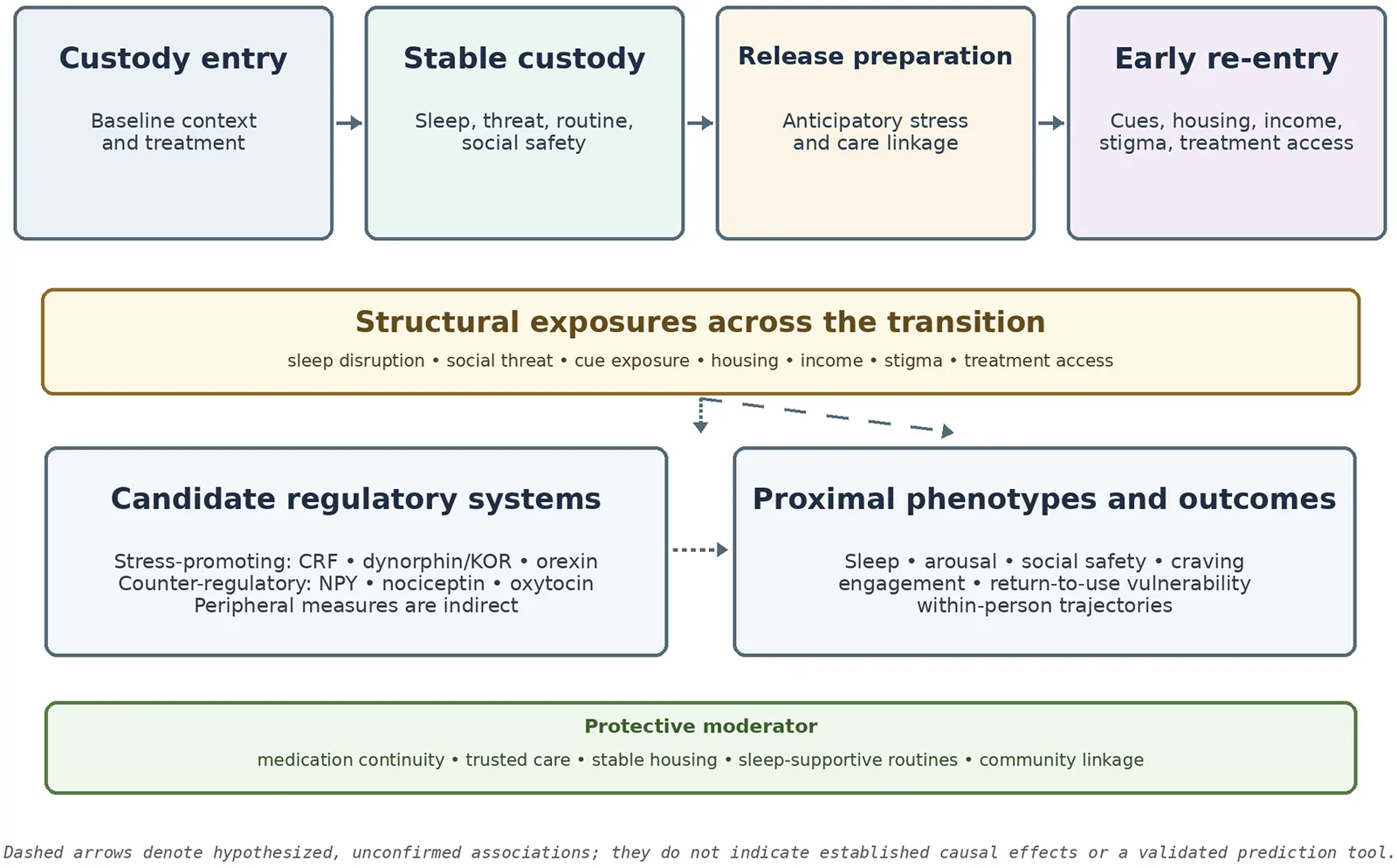
Hypothesized associations across custody, release preparation, and community re-entry. Structural exposures may modify candidate neuropeptide measures and proximal phenotypes; peripheral concentrations are indirect and do not establish central signaling. Dashed arrows denote unconfirmed associations. Continuity of medication, trusted care, stable housing, sleep-supportive routines, and community linkage is proposed as a protective moderator. CRF, corticotropin-releasing factor; KOR, kappa opioid receptor; NPY, neuropeptide Y.

Figure 1 summarizes the framework. Within-person changes may be more informative than a single group average, and medication continuity and trusted care may attenuate adverse trajectories. No peptide panel should be considered clinically useful unless it adds reproducible predictive value beyond contextual conditions, symptoms, and treatment continuity.

Three potential uses must remain distinct. A mechanistic correlate may illuminate a pathway without predicting an individual outcome. A prognostic biomarker must add validated predictive value beyond available clinical and social information. A treatment-response biomarker must identify differential benefit from a defined intervention. Candidate neuropeptides are far from satisfying the latter two standards. The immediate question is whether coordinated, context-linked trajectories are reproducible—not whether a threshold can classify an individual.

## Falsifiable predictions

The model yields four prespecified predictions. First, within-person changes in stress-regulatory phenotypes will cluster around defined transitions—entry, stable custody, release preparation, and re-entry—rather than remain static. Second, greater exposure to sleep disruption, social threat, cue exposure, or treatment interruption will be associated with less favorable trajectories, whereas medication continuity and trusted care will attenuate them. Third, repeated measures will explain more variation in proximal outcomes than a single pre-release value. Fourth, the construct will be weakened if standardized assays show no reproducible transition-related pattern or no incremental information beyond contextual and clinical measures.

## Testing the transition without overinterpreting it

An informative design would follow participants at custody entry, during stable custody, before release, within 72 h after release where feasible, during weeks 1–2, and at days 30 and 90. Investigators should prespecify the specimen matrix and standardize collection time, fasting and medication status, extraction, storage, freeze–thaw exposure, batch allocation, limits of detection, and blinded duplicate assays. Biological observations should be paired with validated measures of sleep, stress, social safety, craving, cue exposure, housing, and treatment continuity.

Analyses should prioritize a small set of primary systems and outcomes, with other tests labeled exploratory. Directed causal diagrams should distinguish time-varying exposures, confounders, mediators, and outcomes. Multilevel models should estimate within-person change; missingness and loss to follow-up require sensitivity analyses; multiplicity requires correction or hierarchical modeling; and any prediction model requires external validation. Altered tolerance, drug availability, infection, nutrition, medication, and acute stress remain competing explanations.

Medication for substance use disorder must never be withheld for mechanistic convenience. Consent must be voluntary and separated from discipline, privileges, parole, release decisions, and access to care. Data governance should specify ownership, access, retention, secondary use, and a prohibition on disclosure to correctional or law-enforcement decision makers. Biospecimens and linked data should not be repurposed for punishment, surveillance, or individualized release prediction. People with lived experience should participate in protocol design, oversight, and interpretation.

A staged program would reduce false discovery. Feasibility studies should establish recruitment, specimen stability, assay reliability, retention, and acceptability. Confirmatory cohorts should preregister directional hypotheses and seek external replication. Only reproducible associations should advance to intervention studies; well-designed null findings would remain informative tests of the model.

## Treatment implications: continuity before precision

The practical priority is continuity, not biomarker-guided care. Evidence-based medication should continue across custody and release [8–10, 12–14]. Sleep stabilization, predictable routines, dependable social connection, naloxone access, housing support, and timely community linkage are justified by clinical and ethical evidence independent of any biomarker. Humane care must never become conditional on a biological result.

Neuropeptide-targeted treatment remains experimental. Nivasorexant entered human trials for non-addiction indications, but efficacy for addiction has not been established [19]. A newer selective orexin-1 antagonist has progressed from preclinical substance-use models toward early human investigation, without clinical efficacy evidence to date [20]. Oxytocin findings also remain inconsistent [18]. The defensible near-term agenda is therefore mechanism-focused research layered onto established medication, housing, harm reduction, and psychosocial care—not a replacement for those interventions.

## Discussion

The carceral allostatic amplifier is deliberately falsifiable. It would be weakened if repeated, standardized measures showed no reproducible transition-related pattern, added no information beyond contextual measures, or failed to track proximal phenotypes after rigorous control for confounding. It would be strengthened by convergence across repeated assays, sleep and behavioral phenotypes, and experimental stress responses. Even convergent peripheral findings would remain correlates unless stronger causal designs supported mediation.

The model offers three contributions: it explains why observed non-use under constraint may overstate recovery; it integrates structural exposure with stress biology without treating either as sufficient; and it redirects biomarker research from static classification toward dynamic transitions. Its limitations are substantial. Human neuropeptide measurement is difficult, translation from animal models is incomplete, correctional environments are heterogeneous, and observational studies are vulnerable to selection, attrition, measurement error, and time-varying confounding.

Feasibility and generalizability will vary across prisons, jails, remand settings, jurisdictions, sexes and genders, racial and ethnic groups, ages, immigration contexts, and lengths of custody. Release timing changes, follow-up is difficult, and health systems differ in medication access, privacy protections, and community linkage. Multisite studies should document these institutional and structural conditions rather than statistically erase them.

We do not advocate neuropeptide screening of incarcerated people. We propose the prison-to-community transition as a rigorous test of when vulnerability changes, for whom, and through which regulatory pathways. One conclusion does not require biomarker validation: pharmacological, relational, and environmental continuity should be preserved across the gate. Neuropeptide science will be useful only if it clarifies how to strengthen that continuity rather than reducing addiction to a molecule.

## Data Availability

No datasets were generated or analysed for this manuscript. The article is based exclusively on previously published literature cited in the manuscript.
